# 2009 Swine-Origin Influenza A (H1N1) Resembles Previous Influenza Isolates

**DOI:** 10.1371/journal.pone.0006402

**Published:** 2009-07-28

**Authors:** Carl Kingsford, Niranjan Nagarajan, Steven L. Salzberg

**Affiliations:** 1 Center for Bioinformatics and Computational Biology, Institute for Advance Computer Studies, University of Maryland, College Park, Maryland, United States of America; 2 Department of Computer Science, University of Maryland, College Park, Maryland, United States of America; University of Birmingham, United Kingdom

## Abstract

**Background:**

In April 2009, novel swine-origin influenza viruses (S-OIV) were identified in patients from Mexico and the United States. The viruses were genetically characterized as a novel influenza A (H1N1) strain originating in swine, and within a very short time the S-OIV strain spread across the globe via human-to-human contact.

**Methodology:**

We conducted a comprehensive computational search of all available sequences of the surface proteins of H1N1 swine influenza isolates and found that a similar strain to S-OIV appeared in Thailand in 2000. The earlier isolates caused infections in pigs but only one sequenced human case, A/Thailand/271/2005 (H1N1).

**Significance:**

Differences between the Thai cases and S-OIV may help shed light on the ability of the current outbreak strain to spread rapidly among humans.

## Introduction

In April 2009, a novel strain of the influenza A (H1N1) virus emerged in Mexico, the United States, and multiple other countries. By early June, the World Health Organization reported that the virus had spread to 66 countries with 19,273 confirmed cases including 117 deaths (http://www.who.int/csr/disease/swineflu/en). In the United States, the Centers for Disease Control had reported 11,054 cases, including 17 deaths, spanning all 50 states. The outbreak strain has been identified as a swine-origin influenza virus that resulted from a reassortment of two previously circulating strains: a “triple-reassortant” swine influenza that has been circulating in North America since 1998 and an H1N1 strain that has been circulating for decades in swine populations in Europe and Asia. The new strain contains six segments from the North American lineage and two segments from the Eurasian lineage [1]. An estimate based on coalescent analysis using 23 public hemagglutinin sequences from distinct locations placed the beginning of the outbreak in early January 2009 [2], although incomplete sampling leads to considerable uncertainty in this date.

## Results

**Figure 1:**
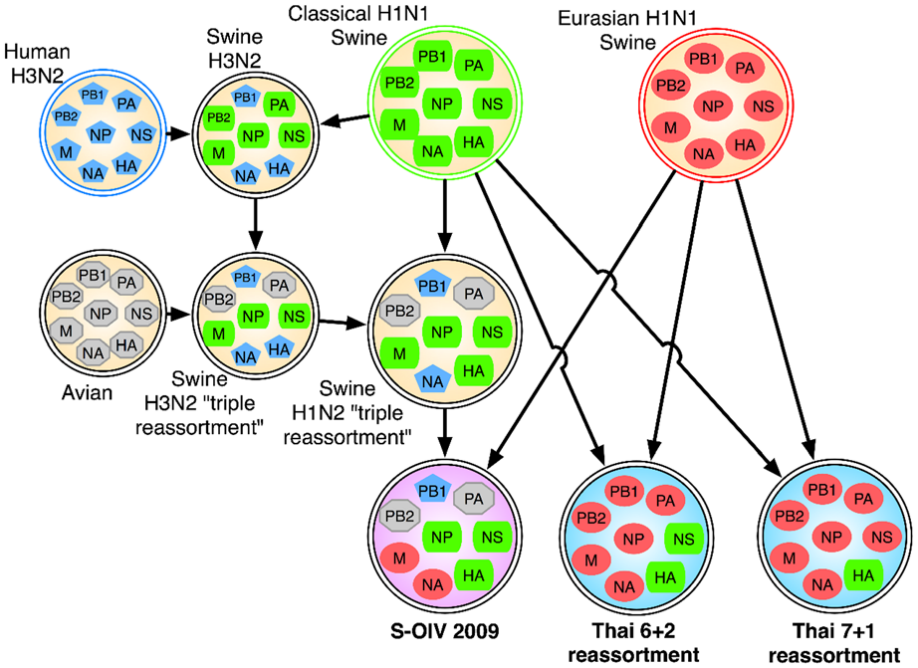
Reassortment history of the 2009 S-OIV outbreak strain and the Thai reassortants. Colors indicate whether the segment derived from human H3N2, classical H1N1 swine, Eurasian H1N1, or avian influenza. The isolates from Thailand represent the only sequenced examples, prior to S-OIV, of reassortment strains containing the HA segment from classical H1N1 swine and the NA segment from the Eurasian H1N1 swine lineage. The reassortment history of the H1N2 “triple reassortment” was described by Olsen [9]. Arrows indicate ancestor relationships; additional, unobserved reassortment events may have occurred.

In this note, we report the results of the application of a comprehensive, computational search for reassortments between the Eurasian and North American swine influenza lineages. Using the genome sequence of one of the isolates from the current S-OIV outbreak, we conducted a comprehensive computational search among all hemagglutinin (HA) and neuraminidase (NA) sequences from H1N1 swine isolates to identify all reassortments matching the outbreak strain. We found that two similar reassortments had occurred among H1N1 isolates collected in Thailand between 2000 and 2006, resulting in multiple infections among pigs and a single sequenced human case, A/Thailand/271/2005 [3], [4]. The human case and two of the swine isolates in Thailand (A/Sw/Chonburi/NIAH9469/2004 and A/Sw/Chonburi/NIAH977/2004) bring together the HA segment from the North American “classical” lineage and the NA segment from the Eurasian lineage, as in the 2009 S-OIV outbreak strain (Figure 1). In the three Thai isolates and the new S-OIV isolates, the segment containing the M protein derives from the Eurasian lineage, and the segment containing the nonstructural protein (NS) derives from the classical lineage. Thus the Thai and S-OIV reassortants have four segments that share similar broad evolutionary history, though there are a number of differences between their sequences. The other four segments — NP, PA, PB1, and PB2 — have a different phylogenetic origin in the Thai sequences than in the 2009 outbreak strain sequences. In the Thai isolates, these internal segments are most closely related to the Eurasian lineage, while in the S-OIV strains they derive from the North American triple-reassortant lineage. The H1N2 triple-reassortant and classical H1N1 lineages share similar HA, M, NS, and NP segments. An additional three isolates, A/swine/Chonburi/05CB1/2005, A/swine/Chonburi/06CB2/2006 [3], [4], and A/swine/Thailand/HF6/2005 [5] have HA and NA segments similar to the Thai sequences described above, but sequences for the internal segments are not available.

A second set of reassortants, also from Thailand, involved only the HA segment (A/Sw/Chonburi/NIAH589/2005, A/Sw/Chachoengsao/NIAH587/2005, A/Sw/Ratchaburi/NIAH550/2003, and A/Sw/Ratchaburi/NIAH1481/2000 [4]). Our analysis reconfirms the previous identification [3], [4], [5], [6] of the two sets of Thai isolates as classical-Eurasian reassortants, while highlighting their importance in the context of the S-OIV strains. As with the first set of Thai strains above, the second set of strains brought a classical-lineage HA into contact with a Eurasian-lineage NA, but these isolates have a Eurasian NS segment in addition to Eurasian-derived NP, PA, PB1, and PB2 segments. Despite extensive recent phylogenetic analyses of swine influenza [1], [2], [7], [11], the similarities in these reassortment architectures remained unreported.

The Thai reassortants were identified here by an automatic, exhaustive, computational search that compares ensembles of trees sampled from a Markov chain Monte Carlo (MCMC) walk. This search, implemented in a novel reassortment discovery program, exhaustively enumerates all HA-NA tree incompatibilities that have sufficient statistical support to be considered likely reassortments despite the ambiguities in phylogenetic reconstruction. Experiments [8] on collections of human, avian, and artificial sequences indicate that the algorithm has a low false-negative rate, suggesting that the Thai sequences listed above, along with the 2009 S-OIV outbreak sequences, are the only sequenced reassortments that bring together classical H1N1 HA segments and Eurasian H1N1 NA segments. Additional reassortments are present among non-H1N1 swine isolates, including, for example, A/Sw/Saraburi/NIAH13021/2005(H1N2), A/Sw/Ratchaburi/NIAH874/2005(H3N2), A/Sw/Nakhon pathom/NIAH586-1/2005(H3N2), A/Sw/Chachoengsao/2003(H3N2), A/Sw/Ratchaburi/NIAH59/2004(H3N2).

**Figure 2:**
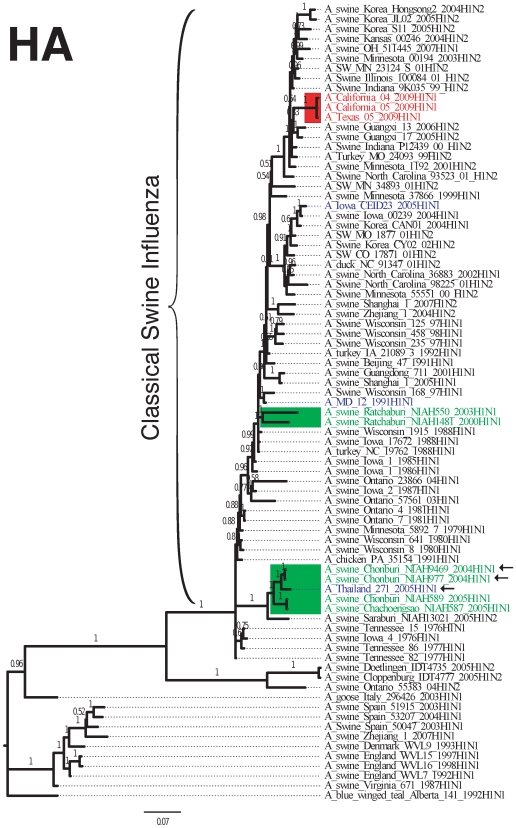
Majority consensus tree for the HA segment computed with MrBayes [14]. Numbers on branches give posterior probabilities. S-OIV (H1N1) 2009 isolates are colored red. To simplify the figure, only three S-OIV isolates are shown; all other sequences from S-OIV are nearly identical and would appear in the same location in the trees. Thai isolates representing the 6+2 and 7+1 reassortments are colored green, with Thai (6+2) reassortants marked by arrows. The human cases of infection with swine-origin influenza that appear in the trees are shown in blue.

**Figure 3:**
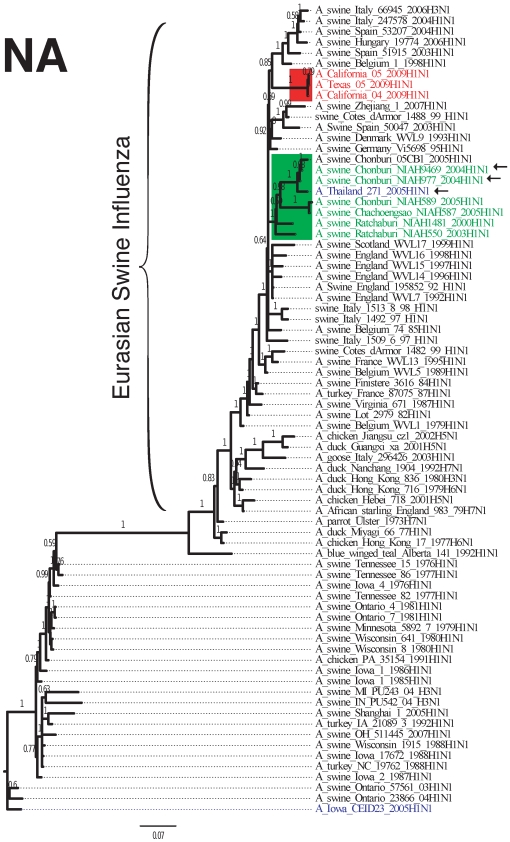
Majority consensus tree for the NA segment, computed and labeled as in Figure 2.

The evolutionary relationship among the S-OIV outbreak strain, the Thai isolates, and other influenza strains is illustrated for the HA and NA segments in Figures 2 and 3. (Trees for the other segments are shown in Figures S1–S6.) The HA tree shows that the most similar sequences to HA for both S-OIV and the Thai reassortants are descendants of classical H1N1, including triple-reassortant H1N2. The sequence similarity between the Thai and S-OIV HA segments ranged from 85–88% for the HA segment and 90–91% for the NA segment. The 7+1 Thai reassortants are split into two clades in the HA tree, probably indicating several reassortment events. Trees for the M, NP, NS, PA, PB1, and PB2 segments are provided in supplemental data.

## Discussion

The evolutionary history of swine influenza A (H1N1) over the past decade is complex. For many decades, H1N1 influenza in North American swine (also called “classical” H1N1) mutated relatively slowly [9], but in 1997, a novel reassortant emerged that contained three segments (HA, NA, and PB1) from the human H3N2 virus, and the remaining five from the classical North American H1N1. Soon thereafter, the first “triple reassortant” viruses were documented [10], in which avian-derived PA and PB2 polymerase genes replaced two of the classical segments in the H3N2 reassortant (Figure 1). These triple reassortants rapidly spread through pig populations, and additional reassortments occurred, including one that created an H1N2 triple reassortant by re-combining the classical HA (H1) segment with an H3N2 triple reassortant (Figure 1). Until the current S-OIV outbreak, there were only a few sporadic cases of human infection by triple-reassortant swine influenza A; the first case in the United States was reported to the Centers for Disease Control in December 2005, and only 11 cases were reported subsequently until February 2009 [11].

The results presented here catalog the complete collection of sequenced reassortments for which a combination of the HA and NA segments similar to the S-OIV outbreak has occurred. Our main novel result is that no other sequenced examples of this pattern besides the discussed Thai isolates could be found. We can confidently say then that among publicly available sequences these isolates represent the complete catalog of such events. The collection shows that this has happened at least twice within the past ten years and that all previous such sequenced reassortments were collected in Thailand. Due to the lack of detailed surveillance of swine populations around the world, this almost certainly represents an underestimate in the frequency of classical-Eurasian reassortment.

Comparisons between these previous reassortant strains and the S-OIV strain may shed light on the cause of S-OIV's virulence. The previous reassortants did not cause a major human outbreak, despite bringing together somewhat similar surface proteins. One hypothesis is that subsequent mutations in the HA or NA proteins was sufficient to facilitate human-to-human transmission of S-OIV. Alternatively, it may be that some combination of the internal proteins not shared with the Thai strains (PA, NP, PB1, and PB2), possibly interacting with changes in the surface proteins, has given S-OIV its ability to cause human outbreaks. Owing to our comprehensive search through the available H1N1 swine isolates, we know the Thai isolates described above represent the complete set of currently available reassortant sequences that are available to answer this question.

## Methods

### Reassortment detection

The automated reassortment finder [8] was run on all HA and NA segments from swine influenza A (H1N1) isolates (167 strains) and 6 S-OIV isolates using a confidence threshold of 0.95. Only the 2009 H1N1 outbreak and Thai strains described above were identified by the exhaustive search as classical-Eurasian reassortants. They passed further statistical tests indicating that they were likely reassortants and were output by the program. Manual inspection of additional trees (constructed as described below) confirmed the reassortments.

### Taxon sampling

All 74390 flu sequences were downloaded from the influenza virus resource (IVR; http://www.ncbi.nlm.nih.gov/genomes/FLU/) on April 29, 2009. CD-HIT [12] was used to produce a non-redundant set of sequences (99% identity threshold). Each segment of A/California/04/2009(H1N1) was aligned against this non-redundant database. For each segment, the top 20 hits for which a whole genome was available (according to IVR) were collected, and all segments contained in these genomes were included. In addition, the top 30 BLAST hits that were not already included (because they lacked whole genomes) were added for each segment. Finally, all available segments from A/California/05/2009(H1N1), A/Texas/05/2009(H1N1), A/Thailand/271/2005(H1N1), A/Sw/Ratchaburi/NIAH1481/2000(H1N1), A/Sw/Ratchaburi/NIAH550/2003(H1N1), A/Sw/Chonburi/NIAH9469/2004(H1N1), A/Sw/Chonburi/NIAH977/2004(H1N1), A/Sw/Chonburi/NIAH589/2005(H1N1), and A/Sw/Chonburi/NIAH587/2005(H1N1) were included if they were not selected via the automated process. This results in a collection of mostly whole genomes (approximately 97 per segment) along with segment-specific close neighbors. For the HA segment, non-H1 sequences were subsequently filtered; similarly, non-N1 sequences were filtered from the NA collection. After initial inspection of neighbor-joining trees, the sequence A/Mink/Nova Scotia/1055488/2007(H3N2) was removed because it often aligned poorly.

### Tree construction

Nucleotide sequences were aligned by the MUSCLE program [13]. Trees were built using the MrBayes [14] MCMC algorithm (GTR model with gamma distributed rate variation among sites, 200000 total iterations, sampling every 200 iterations after a burnin of 100000 iterations). Majority consensus trees were drawn with the FigTree program (http://tree.bio.ed.ac.uk/software/figtree).

## Supporting Information

Figure S1 Tree for the internal M segment, created as described in the main text. Recent S-OIV isolates are colored red, the Thai 6+2 and 7+1 isolates (described in the main text) are colored green. Red and green boxes draw attention to the clades containing most of the S-OIV and Thai H1N1 reassortant sequences. Human isolates that appear in the trees are colored blue. Because sequences with ≥99% sequence identity were filtered out, some human cases of swine-derived influenza are not shown in the trees.
(3.70 MB TIF)

Figure S2 Tree for the internal NP segment, created as described in the main text, and colored as described in the caption of Supplementary Figure S1.
(3.17 MB TIF)

Figure S3 Tree for the internal NS segment, created as described in the main text, and colored as described in the caption of Supplementary Figure S1.
(2.90 MB TIF)

Figure S4 Tree for the internal PA segment, created as described in the main text, and colored as described in the caption of Supplementary Figure S1.
(3.69 MB TIF)

Figure S5 Tree for the internal PB1 segment, created as described in the main text, and colored as described in the caption of Supplementary Figure S1.
(3.83 MB TIF)

Figure S6 Tree for the internal PB2 segment, created as described in the main text, and colored as described in the caption of Supplementary Figure S1.
(3.88 MB TIF)
