# Supplementary figures and images for: 2009 Swine-Origin Influenza A (H1N1) Resembles Previous Influenza Isolates
Source: PLoS One. 2009 Jul 28;4(7):e6402. doi: 10.1371/journal.pone.0006402 (PMC2712239; doi:10.1371/journal.pone.0006402)

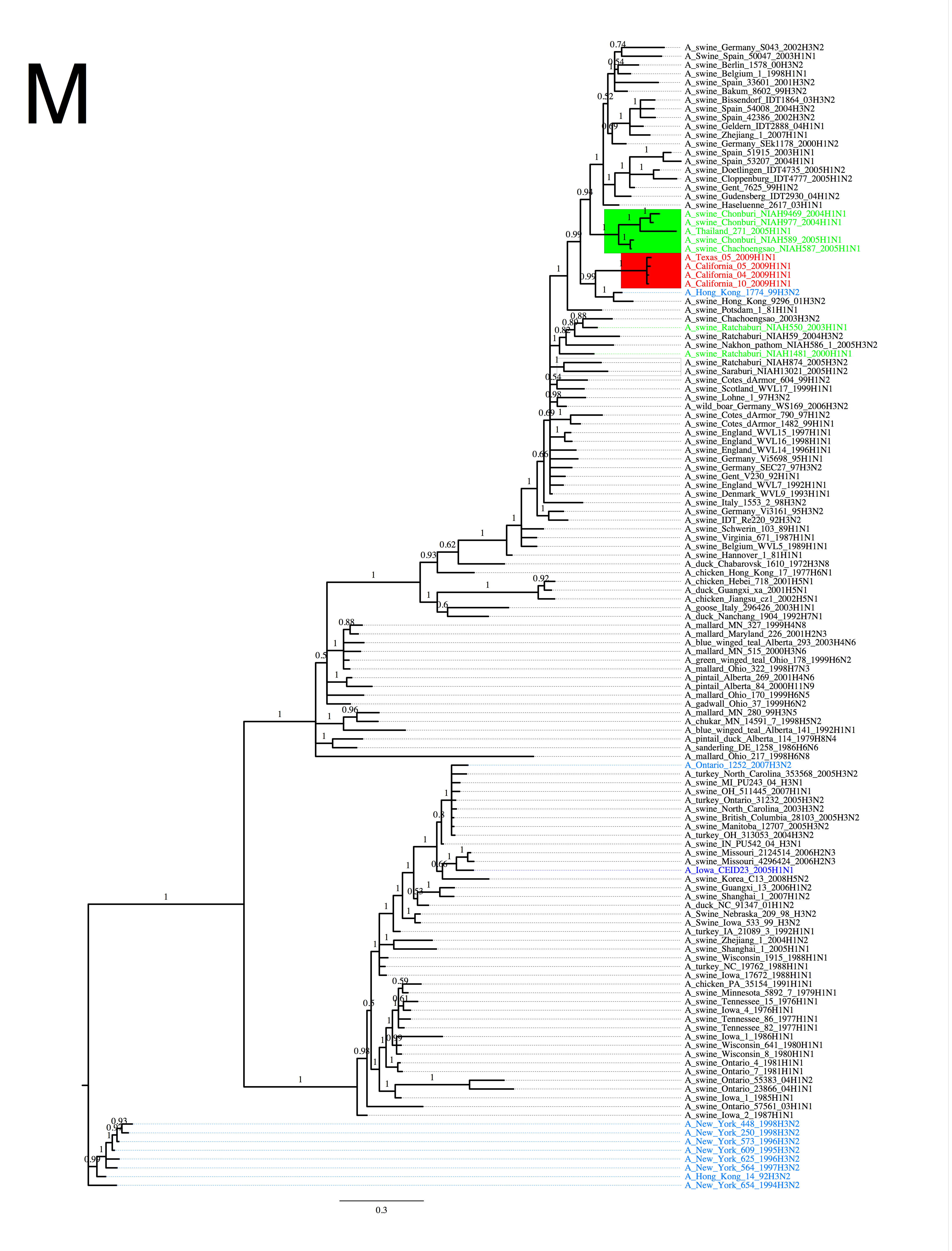

Supplement: Figure S1 — Tree for the internal M segment, created as described in the main text. Recent S-OIV isolates are colored red, the Thai 6+2 and 7+1 isolates (described in the main text) are colored green. Red and green boxes draw attention to the clades containing most of the S-OIV and Thai H1N1 reassortant sequences. Human isolates that appear in the trees are colored blue. Because sequences with ≥99% sequence identity were filtered out, some human cases of swine-derived influenza are not shown in the trees. (3.70 MB TIF) [file pone.0006402.s001.tif]

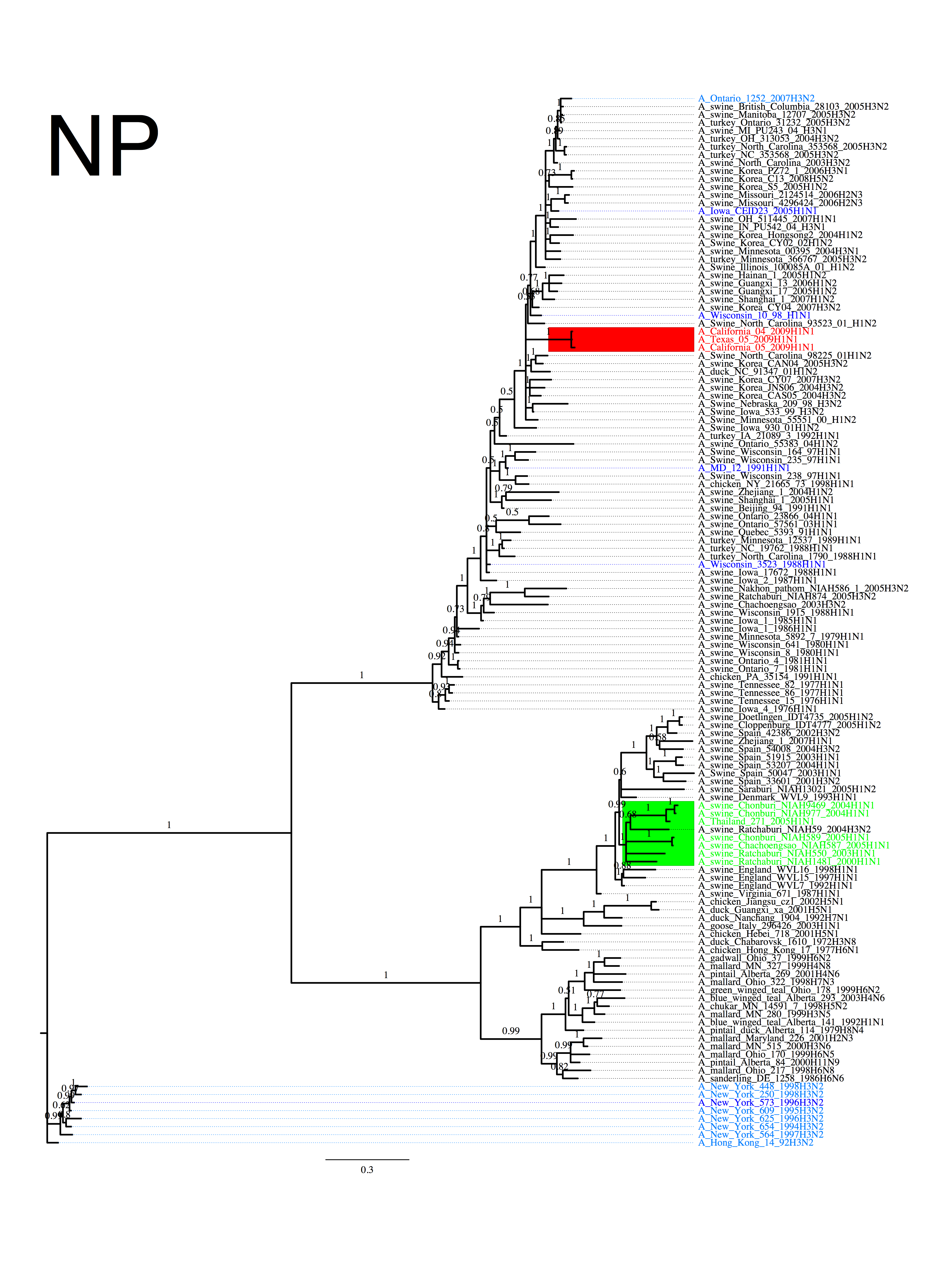

Supplement: Figure S2 — Tree for the internal NP segment, created as described in the main text, and colored as described in the caption of Supplementary Figure S1. (3.17 MB TIF) [file pone.0006402.s002.tif]

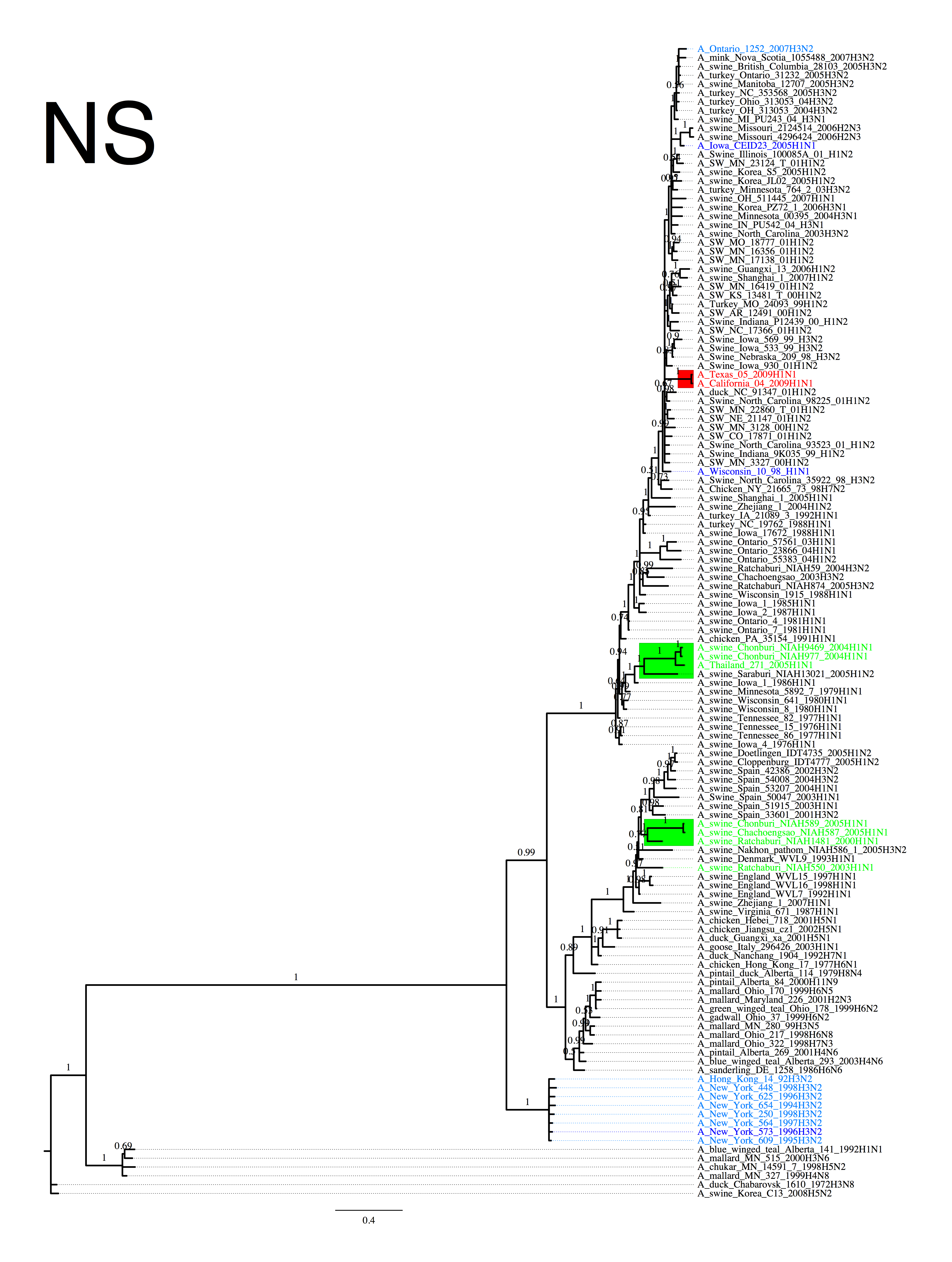

Supplement: Figure S3 — Tree for the internal NS segment, created as described in the main text, and colored as described in the caption of Supplementary Figure S1. (2.90 MB TIF) [file pone.0006402.s003.tif]

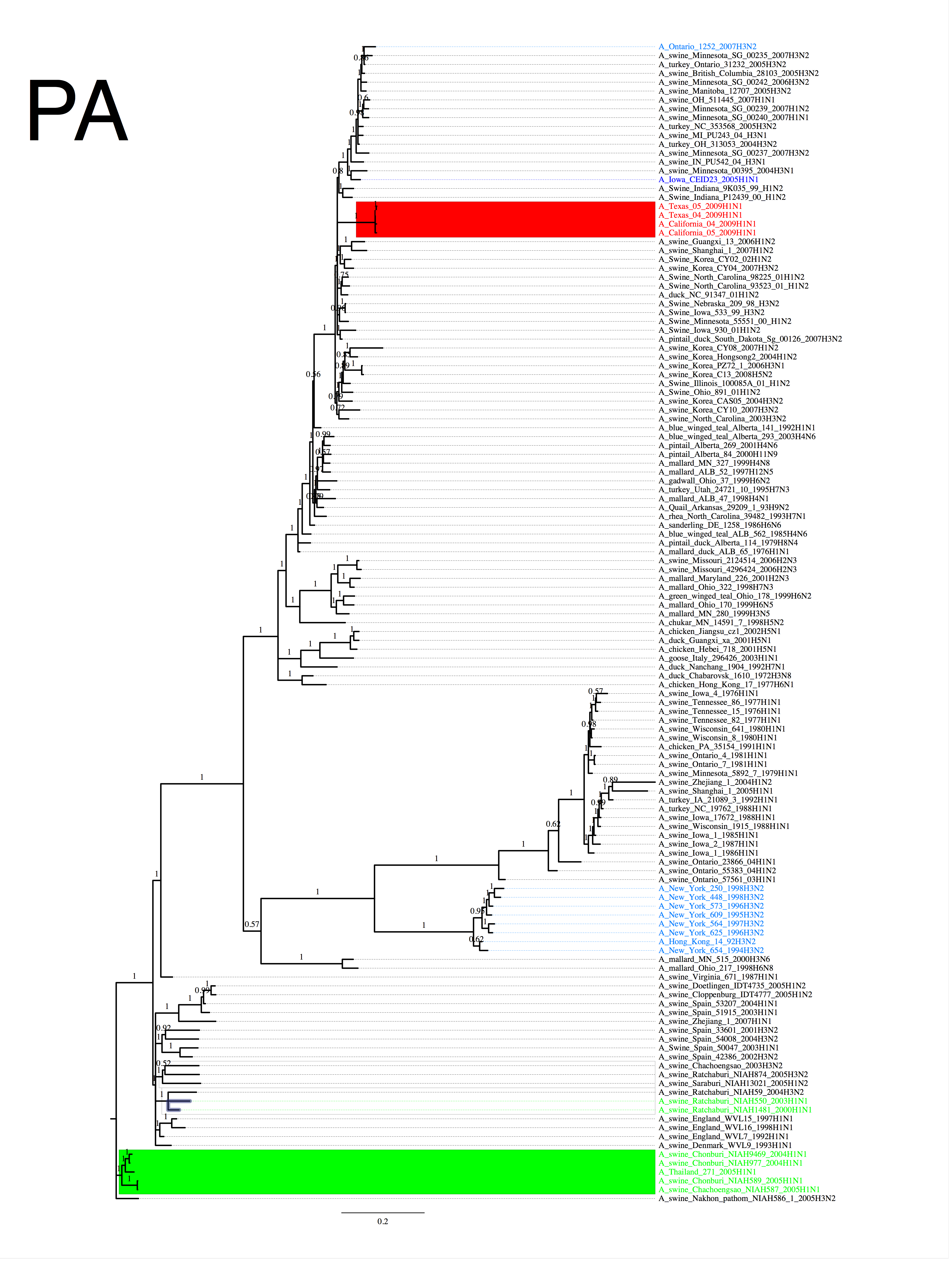

Supplement: Figure S4 — Tree for the internal PA segment, created as described in the main text, and colored as described in the caption of Supplementary Figure S1. (3.69 MB TIF) [file pone.0006402.s004.tif]

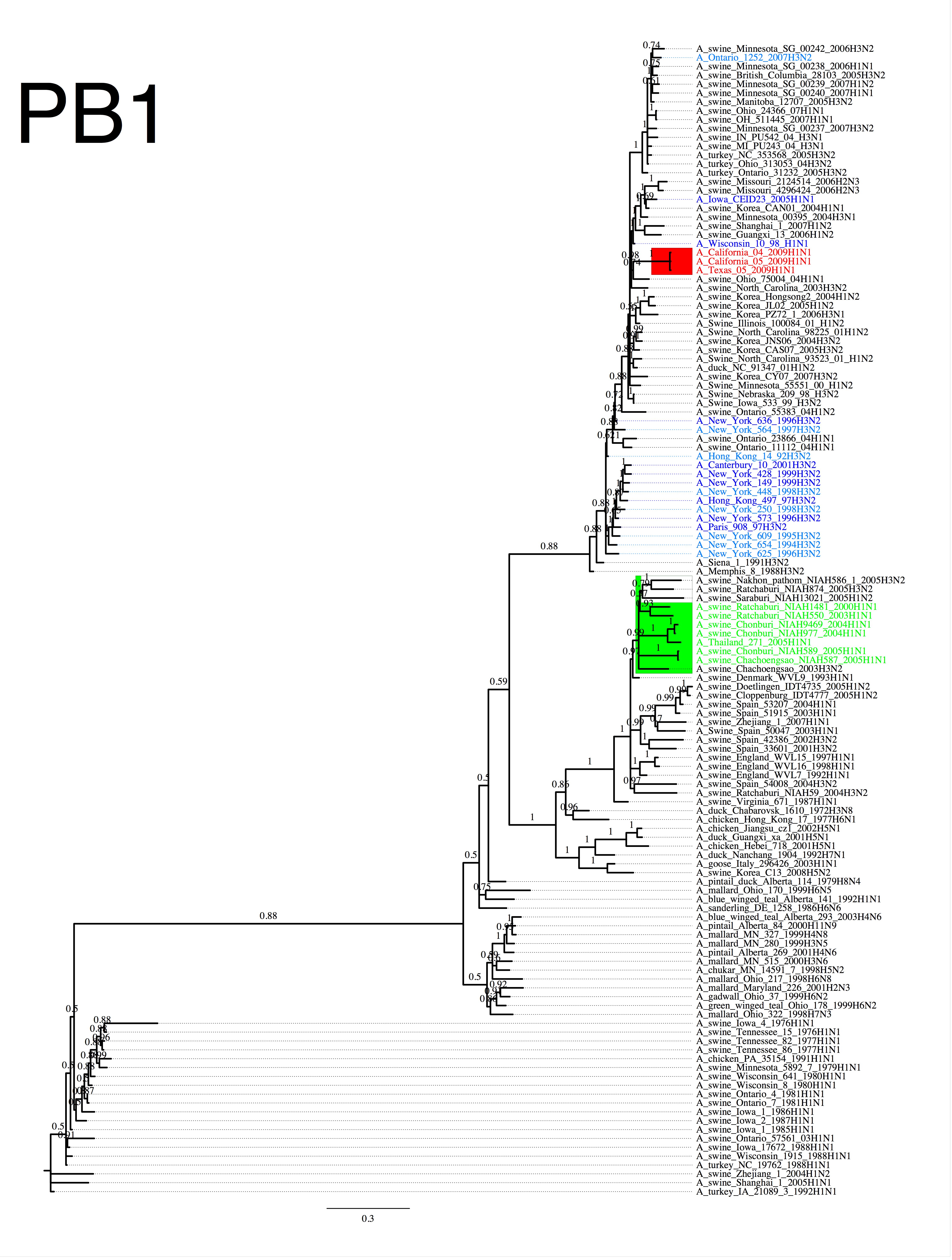

Supplement: Figure S5 — Tree for the internal PB1 segment, created as described in the main text, and colored as described in the caption of Supplementary Figure S1. (3.83 MB TIF) [file pone.0006402.s005.tif]

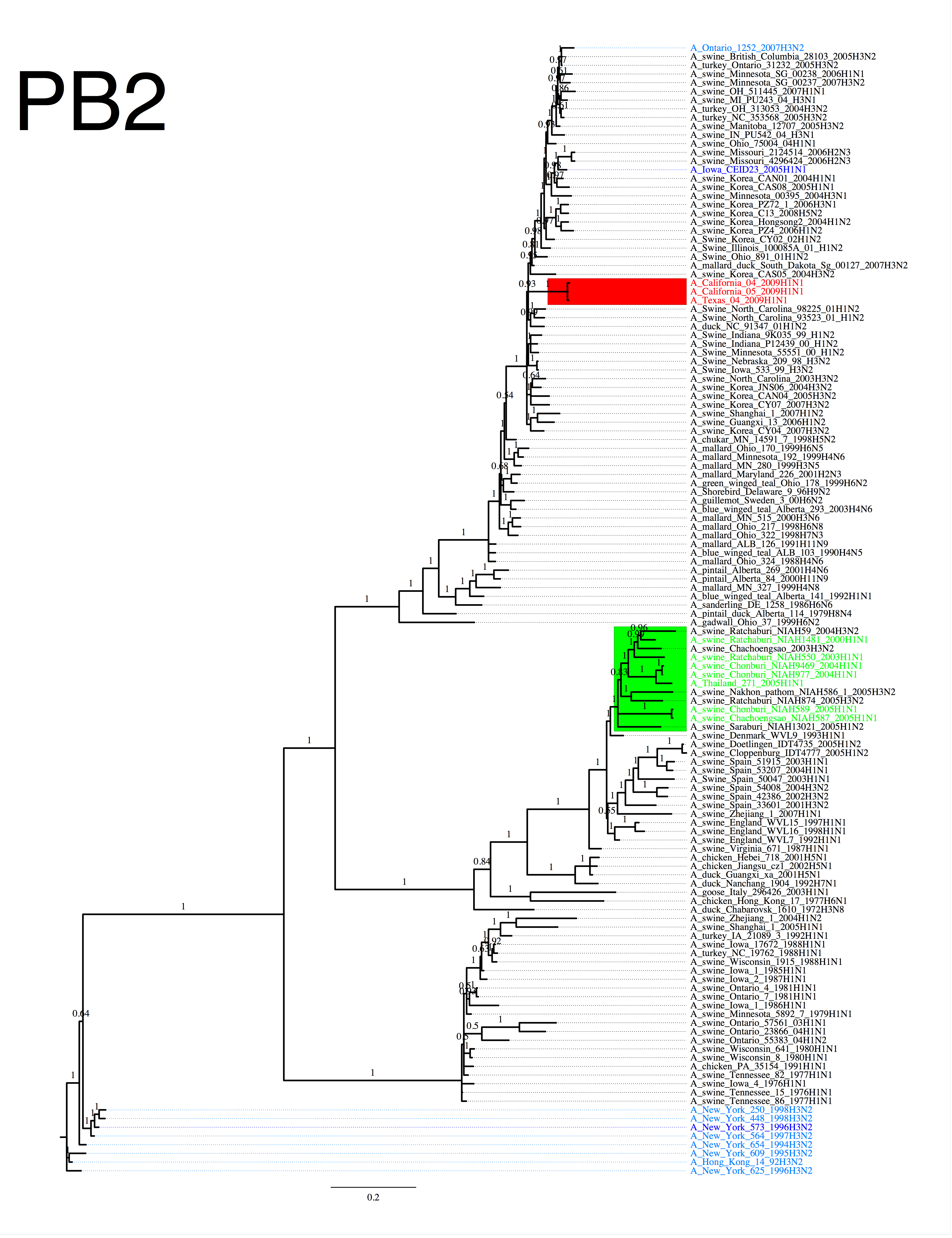

Supplement: Figure S6 — Tree for the internal PB2 segment, created as described in the main text, and colored as described in the caption of Supplementary Figure S1. (3.88 MB TIF) [file pone.0006402.s006.tif]
